# Current role of trisectionectomy for hepatopancreatobiliary malignancies

**DOI:** 10.1002/ags3.12292

**Published:** 2019-10-22

**Authors:** Philipp Kron, Norihisa Kimura, Shahid Farid, J. Peter A. Lodge

**Affiliations:** ^1^ Department of HPB and Transplant Surgery St. James's University Hospital Leeds UK

**Keywords:** colorectal liver metastases, liver resection, right hepatic trisectionectomy

## Abstract

**Background:**

Trisectionectomy is a treatment option in extensive liver malignancy, including colorectal liver metastases (CRLM). However, the reported experience of this procedure is limited. Therefore, we present our experience with right hepatic trisectionectomy (RHT) for CRLM as an example and discuss the changing role of trisectionectomy in the context of modern treatment alternatives based on a literature review.

**Methods:**

Between January 1993 and December 2014 all patients undergoing RHT at a single center in the UK for CRLM were included. Patient and tumor characteristics were reviewed and a multivariate analysis was done. Based on a literature review the role of trisectionectomy in the treatment of HPB malignancies was discussed.

**Results:**

A total of 211 patients undergoing RHT were included. Overall perioperative morbidity was 40.3%. Overall 90‐day mortality was 7.6% but reduced to 2.8% over time. Multivariate analysis identified additional organ resection (*P* = .040) and blood transfusion (*P* = .028) as independent risk factors for morbidity. Multiple tumors, total hepatic vascular exclusion, and R1 resection were independent risk factors for significantly decreased disease‐free and disease‐specific survival. Further surgery for recurrence after RHT significantly prolonged survival compared with palliative chemotherapy only.

**Conclusion:**

With the further development of surgical and multimodal treatment strategies in CRLM the indications for trisectionectomy are decreasing. Having being formerly associated with high rates of perioperative morbidity and mortality, this single‐center experience clearly shows that these concomitant risks decrease with experience, liberal use of portal vein embolization and improved patient selection. Trisectionectomy remains relevant in selected patients.

## Abstract 2

Systematic review on the use of trisectionectomies in the treatment of HPB malignancies.

## Graphical abstract

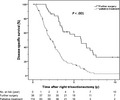

## 1 INTRODUCTION

Left hepatic trisectionectomy (LHT) was first described in detail by Starzl and colleagues as a left trisegmentectomy in 1982, and then as an extended left hepatectomy by Blumgart et al in 1993.^1^, ^2^ The designation LHT was adopted following the International Hepato‐Pancreato‐Biliary Association Brisbane 2000 consensus statement on the nomenclature of liver anatomy and resection. LHT is defined as excision of Couinaud liver segments 2, 3, 4, 5 and 8, with or without segment 1.^3^ Despite improvements in surgical techniques and perioperative patient management, only a few papers have reported outcomes of LHT in more than 10 patients.^4^, ^5^, ^6^, ^7^, ^8^ Morbidity and mortality after LHT is higher than for other hepatectomies, and this procedure is reserved for patients with a significant tumor burden and an otherwise dismal prognosis. The high morbidity rate is attributable mainly to the aggressive nature of the disease being treated, but may also be related to the extent of liver volume resected, estimated to be as high as 80 per cent.^2^ In 2005, the Leeds group reported long‐term outcomes of LHT in 70 consecutive patients.^4^ Morbidity rate was high, but the potential for cure supported an aggressive surgical resection policy where other treatment options had been exhausted. In 2016, the same group described changes in surgical practice over time, and analyzed the short‐ and long‐term outcomes of LHT for hepatobiliary malignancy, in order to identify factors associated with morbidity and mortality in the modern era.^9^

Right hepatic trisectionectomy (RHT) was first described by Lortat‐Jacob, Robert and Henry as right lobectomy in 1952.^10^ This operation has had a number of different names, but, until recently, it has been most commonly known as right trisegmentectomy. The designation RHT was adopted following the International Hepato‐Pancreato‐Biliary Association Brisbane 2000 consensus statement on the nomenclature of liver anatomy and resection.^3^, ^11^ This procedure requires excision of segments 4, 5, 6, 7 and 8 ± 1 and it also remains one of the most challenging major hepatectomies. Despite improvements in surgical technique and perioperative critical management, perioperative morbidity remains high and only a few hepatobiliary centers worldwide have reported their experience.^12^, ^13^

Modifications of LHT and RHT by in‐contiguity and non‐anatomical extension and repeat liver resection after LHT or RHT are also rarely reported.^14^, ^15^

The role of these technically demanding and extensive resections in contemporary hepatobiliary practice is established for primary liver cancers and for those tumors with no significant neoadjuvant strategies, but it is also changing as new treatments emerge. This is particularly true for patients with colorectal liver metastases (CRLM), and it is likely that this trend will be followed for other HPB malignancies as more effective preoperative strategies are developed. Emerging data for intrahepatic cholangiocarcinoma, for example, is encouraging.^16^, ^17^, ^18^, ^19^

For patients with CRLM, despite the lack of compelling data for most patients, there has been a paradigm shift in the oncological assessment of patients and the use of neoadjuvant and “downstaging” strategies before resection. This has been combined with a sensible development of surgical strategies aimed at parenchymal preservation, along with new developments in liver surgery such as multistage resection as a classical two‐stage approach (TSH) or associating liver partition and portal vein ligation for staged hepatectomy (ALPPS). In the classical two‐stage approach, portal vein embolization (PVE) or portal vein ligation (PVL) is carried out to stimulate hypertrophy in the planned future liver remnant, along with resection of tumors from the planned future liver remnant (FLR). After an interval of 4‐8 weeks, with adequate hypertrophy of the FLR, the definitive resection is carried out.^20^ Besides PVL/PVE, the first step in ALPPS includes at least a 50% transection of liver parenchyma.^21^ By this modification, ALPPS seems to be able to accelerate liver growth of the FLR and to shorten the interstage interval.^22^, ^23^ A recent Scandinavian randomized controlled trial has shown the benefits of ALPPS in providing a higher resection rate compared to the classical two‐stage procedure, with comparable margins, complications and short‐term mortality.^24^

However, besides the evolvement of these promising strategies, there remains a place for up‐front major resection for many patients. In the light of this trend, we have reviewed in detail a 22‐year single‐center experience of RHT for CRLM and evaluated factors affecting morbidity and survival in order to provide a critical appraisal for the role of RHT for CRLM in order to add these data to our previous work on LHT.

## 2 METHODS

### 2.1 Study design

Patients undergoing RHT between January 1993 and December 2014 were identified from a prospectively maintained database at a single institution. Additional data from the database included radiological investigations and interventions, presence or absence of jaundice, extent of surgical resection, duration of operation, requirement for transfusion of blood or blood products, need for Pringle maneuver or total vascular exclusion, additional surgery (lymphadenectomy, extrahepatic bile duct excision with reconstruction, or vascular reconstruction), histopathological diagnosis, size and distribution of tumors, perioperative morbidity and mortality, and long‐term disease‐free and disease‐specific survival. This work has been reported in line with the PROCESS criteria.^25^

All patients undergoing liver resection were offered adjuvant chemotherapy according to guidelines unless they had received adjuvant therapy following their colonic resection within the past 12 months. However, detailed data on adjuvant chemotherapy after colorectal and hepatic surgery were not routinely collected in the database owing to the large number of patients presenting from a wide geographical area of referring hospitals using chemotherapy. In 12 patients, neoadjuvant chemotherapy was used as either a downsizing technique or as a “test of time approach.”

### 2.2 Preoperative evaluation

Preoperative radiological assessment in all patients included thoracic, abdominal and pelvic computed tomography (CT), and magnetic resonance imaging (MRI) of the liver. The investigations were reviewed in a multidisciplinary team meeting to discuss and define the extent of resection. In selected cases, positron emission tomography CT (PET‐CT) was used. From 2007, PVE was used when the future liver remnant was estimated to be <20% and was carried out 3 to 4 weeks before scheduled liver resection, but no formal volumetry studies have been done in our center.

### 2.3 Perioperative care

Techniques of RHT and extensions of RHT have been described previously.^14^, ^26^, ^27^ Intraoperative ultrasound was carried out in all patients to identify any additional lesions in segments 2 and 3, and their relation to the left portal structures and hepatic veins. All liver transections were carried out using a Cavitron ultrasonic surgical aspirator (CUSA). Pringle's maneuver was applied in selected patients to reduce blood loss and total hepatic vascular exclusion (TVE) (portal triad and hepatic vein or inferior vena cava [IVC] clamping) was used when necessary for tumors located at the hepatocaval confluence. Intraoperative allogeneic red blood cells (ARBC) and fresh frozen plasma were transfused at the discretion of the anesthesiologist. ARBC were also transfused postoperatively if the hemoglobin level fell to <8.0 g/dL in the absence of cardiac disease and <10.0 g/dL for patients with risk factors for cardiac disease according to our unit policy. No patients received autologous blood transfusion.

### 2.4 Morbidity and mortality

Details of complications were obtained from the database and, where necessary, from the patient notes and graded according to the validated Clavien‐Dindo classification system.^28^ Postoperative liver failure was defined according to the International Study Group of Liver Surgery.^29^ Postoperative mortality was defined by the occurrence of death within 90 days of surgery or at any time during postoperative hospital stay.

### 2.5 Histopathological evaluation

Pathological reports were reviewed to determine tumor histological grade, margin status, and histological abnormalities in the non‐tumor‐bearing liver (NTBL). A tumor‐free resection margin of less than 1 mm was classified as (R1), and 1 mm or more was classified as (R0).^30^ In relation to NTBL, liver steatosis was defined as diffuse accumulation of fat droplets affecting >5% of hepatocytes.^31^ Fibrosis was scored according to the Metavir score, and defined as the presence of portal fibrosis with/without septa, numerous septa, or cirrhosis.^32^ Sinusoidal injury was graded and defined as the presence of centrilobular involvement beyond one‐third of the lobular area.^33^ These findings in NTBL were defined as parenchymal liver damage in the present study.

### 2.6 Follow up

All patients were followed up regularly at the outpatient clinic at 1, 3, 6 and 12 months in the first year, 18 and 24 months in the second year, and yearly thereafter if the patient remained disease‐free. Follow up included clinical examination and assessment of tumor markers (carcinoembryonic antigen [CEA], cancer antigen [CA]19‐9). Surveillance imaging included CT scans of the chest, abdomen, and pelvis at 3, 6, 12, 18 and 24 months, annually to 5 years and again at 7 and 10 years. MRI and PET‐CT were carried out if recurrence was suspected in routine follow up.

### 2.7 Survival

Disease‐free survival (DFS) was defined as the time from operation to the first documented disease recurrence on imaging. Disease‐specific survival (DSS) was defined as the time from operation to the time of death as a result of recurrence or the most recent follow‐up time. Patients dying of other causes with no evidence of recurrence were censored. In this study of effect on long‐term disease and survival, patients with death within 90 days of operation were excluded.

### 2.8 Statistical analysis

Continuous variables were expressed as median and interquartile range. To consider changes over the study period, patients were divided into three periods based on time interval of treatment: time period 1, 1993‐2000; time period 2, 2001‐2007; time period 3, 2008‐2014. The Kruskal‐Wallis test was used for continuous variables and the Pearson chi‐squared test or Fisher's exact test, where appropriate, for categorical variables. Univariate analysis for postoperative complications was carried out using the Pearson chi‐squared test or Fisher's exact test where appropriate. Multivariate analysis was carried out by Cox regression (stepwise forward model) for variables shown to have a significant influence on postoperative morbidity, 90‐day mortality, and disease‐specific overall and disease‐free survival in the univariate analysis. Date of last follow up was February 2015. All statistical analyses were done using SPSS for Windows/MacTM version 20.0 (IBM), and statistical significance was taken at the 5% level.

## 3 RESULTS

### 3.1 Patient characteristics

Between January 1993 and December 2014, a total of 3946 liver resections were carried out at this single UK center. Of these, 399 (10%) patients underwent RHT, of whom 188 (47%) patients (hepatocellular carcinoma, n = 35 (18.5%); non CRLM, n = 31 (16.5%); hilar cholangiocarcinoma, n = 36 (19%); intrahepatic cholangiocarcinoma, n = 20 (11%); gallbladder cancer, n = 16 (8.5%); benign liver tumor, n = 15 (8%); other malignant liver tumor, n = 6 (3.2%); other benign bile duct disease, n = 5 (2.7%); RHT as part of auxiliary orthotopic liver transplantation, n = 24 (12.8%) were excluded.

A total of 211 patients were included: 126 (60%) male, 85 (40%) female with a median age of 62 years (range, 25‐85). All of the 211 patients included in this analysis underwent RHT for CRLM.

### 3.2 Tumor characteristics

In the cohort, 49 patients (23%) had solitary tumors and median size of the largest tumor was 50 (range 8‐410) mm. Neoadjuvant chemotherapy was given to 12 (5.6%) patients. In 13 (6%) patients, portal vein embolization was done before the actual surgery. Twenty‐five (12%) and 80 (38%) patients underwent concomitant segment (S)1 and/or S2/S3 metastectomy, respectively. Three (1.4%) patients underwent ex vivo resection. Thirty‐two (15%) patients required additional organ resection: in 19 (59%) patients, the diaphragm had to be resected, followed by large bowel n = 8 (25%) and others n = 5 (16%). In 72 (34%) of the patients, some form of parenchymal liver damage was noted: 57 (27%), six (3%) and 18 (9%) with steatosis, fibrosis and sinusoidal obstructive syndrome, respectively. Some patients had two or three duplicate types of liver damage.

### 3.3 Short‐ and long‐term outcomes, morbidity and mortality

Forty‐four patients (21%) received a blood transfusion, with a median ARBC transfusion of 4 units (range 1‐40). Median hospital stay was 10 days (range, 4‐139 days). Of the 211 patients who underwent RHT for CRLM, 85 (40.3%) had postoperative complications as described in Table 1. Thirty‐eight (18%) patients had more than two postoperative complications. Re‐laparotomy was carried out in 19 (9%) patients of the cohort, the main reason being intra‐abdominal bleeding n = 9 (47.4%). Four (21%) of the patients who underwent re‐laparotomy died in hospital.

**Table 1:** Postoperative outcomes after right trisectionectomy for colorectal liver metastases

Outcomes	n (%)
Overall morbidity	85 (40.3)
Grade I	10 (4.7)
Grade II	21 (10.0)
Grade IIIa	11 (5.2)
Grade IIIb	13 (6.2)
Grade IVa	7 (3.3)
Grade IVb	8 (3.8)
Grade V (in‐hospital death)	15 (7.1)
Median hospital stay, days (range)^a^	10 (4‐139)
90‐d mortality	16 (7.6)
Morbidity details^b^	
Transient liver failure	25 (11.8)
Wound infection	12 (5.7)
Bile leak	12 (5.7)
Sepsis	11 (5.2)
Intra‐abdominal bleeding	11 (5.2)
Renal failure	9 (4.3)
Pneumonia	8 (3.8)
Cardiac events^c^	6 (2.8)
Gastrointestinal bleeding	6 (2.8)
Intra‐abdominal fluid collection	6 (2.8)
Wound dehiscence	3 (1.4)
Intra‐abdominal abscess	2 (0.9)
Bowel obstruction	2 (0.9)
Pulmonary embolism	2 (0.9)
Portal vein thrombosis	2 (0.9)
Minor non‐specific complications	9 (4.3)

a Excluding 15 patients who died in hospital.

b 38 patients had two or more complications.

c Including myocardial infarction, congestive heart failure, and arrhythmia.

Of the whole cohort, 15 (7.1%) patients died in hospital. One other patient (0.5%) died within 90 days following surgery. Therefore, 16 patients (7.6%) died within 90 days. Among these 16 patients, main causes for mortality were as follows: seven (44%) patients died from multi‐organ failure; three (19%) patients died as a result of gastrointestinal bleeding; two (13%) due to acute myocardial infarction; one (6%) as a result of pneumonia or intra‐abdominal abscess (n = 1, 6%); massive abdominal bleeding (n = 1, 6%) in hospital; and unknown cause (n = 1, 6%) after discharge. The 211 included patients were further divided into three time periods where 70 patients were included in the first period, 70 patients in the second period and 71 patients in the third period, respectively. With increasing experience at our center we were able to decrease 90‐day mortality from 12.8% to 7.1% and 2.8% accordingly. These differences were not significant.

Univariate analysis for morbidity showed that other organ resection (*P* = .017) and ARBC transfusion (*P* = .012) were markers for poor outcome. Both variables were also found to be independent predictors for morbidity in multivariate analysis (odds ratio [OR] for other organ resection = 2.27; 95% confidence interval [CI], 1.04‐4.95; *P* = .040) and ARBC transfusion (OR = 2.16; 95% CI, 1.09‐4.30; *P* = .028). Notably, parenchymal liver damage in NTBL did not significantly impact morbidity, disease recurrence and survival (Table 2). Median follow up was 29.8 months (range, 0 to 255.7 months) in the entire cohort. DSS rates at 1, 3, 5, and 10 years after RHT were 89.7%, 55.7%, 33.7%, and 22.4%, respectively, with a median DSS of 39.7 months. One‐, 3‐, and 5‐year DFS were 58.7%, 26.6%, and 20.9%, respectively, with median DFS time of 13.3 months. Significant predictors of decreased DFS in univariate analysis were preoperative PVE (*P* = .030), preoperative chemotherapy (*P* = .029), multiple tumors (*P* < .001), caudate lobectomy (*P* = .034), additional metastectomy from segment 2 and/or 3 (*P* < .001), other organ resection (*P* = .015), TVE (*P* = .001), and R1 resection (*P* < .001). In multivariate analysis, multiple tumors (risk ratio [RR] = 1.99; 95% CI, 1.24‐3.20; *P* = .005), TVE (RR = 3.05; 95% CI, 1.42‐6.57; *P* = .004), and R1 resection (RR = 1.60; 95% CI, 1.11‐2.29; *P* = .012) were independent prognostic factors for DFS (Table 3). Likewise, for DSS, preoperative PVE (*P* = .009), preoperative chemotherapy (*P* = .043), multiple tumors (*P* < .001), combined caudate lobectomy (*P* = .042), partial resection of segment 2 and/or 3 (*P* = .023), TVE (*P* = .009), and R1 resection (*P* < .001) were found to be significant factors. In multivariate analysis, multiple tumors (RR = 2.95; 95% CI, 1.68‐5.20; *P* < .001), TVE (RR = 3.77; 95% CI, 1.73‐8.20; *P* = .001), and R1 resection (RR = 1.92; 95% CI, 1.31‐2.81; *P* < .001) were independent prognostic factors for DSS as with DFS (Table 4).

**Table 2:** Univariate and multivariate analyses of variables affecting morbidity after right trisectionectomy

Variables	Total	Morbidity (%)	Univariate analysis		Multivariate analysis	
	n = 211	n = 85 (40.3)	Odds ratio (95% CI)	*P*‐value	Odds ratio (95% CI)	*P*‐value
Preoperative variables						
Gender						
Female	85	32 (37.6)	1.20 (0.68‐2.11)	.521		
Male	126	53 (42.1)				
Age						
≤70 y	165	63 (38.2)	1.48 (0.77‐2.87)	.238		
>70 y	46	22 (47.8)				
Preoperative PVE						
No PVE	198	79 (39.9)	1.29 (0.42‐3.99)	.656		
PVE	13	6 (46.2)				
Preoperative chemotherapy						
No	122	49 (40.2)	1.01 (0.58‐1.77)	.967		
Yes	89	36 (40.4)				
Size of largest tumor						
≤100 mm	170	73 (42.9)	0.55 (0.26‐1.15)	.806		
>100 mm	41	12 (29.3)				
No. of tumors						
Solitary	49	19 (38.8)	1.09 (0.56‐2.09)	.806		
Multiple	162	66 (40.7)				
Parenchymal liver damage^a^						
No	139	58 (41.7)	0.84 (0.47‐1.50)	.553		
Yes	72	27 (37.5)				
Intra‐ and postoperative variables						
S1 resection						
No	186	71 (38.2)	2.06 (0.89‐4.79)	.088		
Yes	25	14 (56.0)				
S2/3 partial resection						
No	131	57 (43.5)	0.70 (0.39‐1.24)	.221		
Yes	80	28 (35.0)				
Other organ resection						
No	179	66 (36.9)	2.50 (1.16‐5.39)	.017*	2.27 (1.04‐4.95)	.040*
Yes	32	19 (59.4)				
Vascular resection^a^						
No	196	76 (38.8)	2.37 (0.81‐6.92)	.106		
Yes	15	9 (60.0)				
Pringle's maneuver						
No	74	30 (40.5)	0.98 (0.55‐1.75)	.956		
Yes	137	55 (40.1)				
Total hepatic vascular exclusion						
No	197	77 (39.1)	2.08 (0.69‐6.22)	.183		
Yes	14	8 (57.1)				
Allogenic red blood cell transfusion						
No	167	60 (35.9)	2.35 (1.20‐4.61)	.012*	2.16 (1.09‐4.30)	.028*
Yes	44	25 (56.8)				
Fresh frozen plasma transfusion						
No	144	57 (39.6)	1.10 (0.61‐1.98)	.761		
Yes	67	28 (41.8)				

PVE, portal vein embolization; S1, caudate lobe; S2/3, left lateral section.

Including steatosis, fibrosis, and sinusoidal obstruction syndrome.

a Including resections of portal vein, hepatic artery, and inferior vena cava.

* *P* < .05.

**Table 3:** Univariate and multivariate analyses of variables predicting disease‐free survival after right trisectionectomy

Variables	Total n = 195^a^	3‐y DFS (%)	MST (months)	Univariate		Multivariate	
				*P*‐value		RR (95% CI)	*P*‐value
Preoperative variables							
Gender							
Female	80	25.1	13.0		.561		
Male	115	27.6	15.4				
Age							
≤70 y	157	26.1	13.1	.348			
>70 y	38	28.2	14.6				
Preoperative PVE							
No PVE	182	28.0	13.9	.030*		1.22 (0.59‐2.51)	.589
PVE	13	0.0	7.5				
Preoperative chemotherapy							
No	111	31.4	18.5	.029*		1.23 (0.87‐1.74)	.246
Yes	84	19.6	11.0				
Neoadjuvant chemotherapy							
No	183	30.0	17.6	.076			
Yes	12	21.6	13.8				
Size of largest tumor							
≤100 mm	158	25.9	13.3	.436			
>100 mm	37	28.9	18.9				
No. of tumors							
Solitary	42	50.5	37.0	<.001*		1.99 (1.24‐3.20)	.005*
Multiple	153	19.9	12.4				
Type of liver metastasis							
Synchronous	107	23.0	13.3	.436			
Metachronous	88	30.3	13.1				
Intra‐ and postoperative variables							
S1 resection							
No	174	28.3	14.6	.033*		1.05 (0.59‐1.85)	.881
Yes	21	11.0	7.4				
S2/3 partial resection							
No	119	33.5	22.6	<.001*		1.40 (0.98‐1.99)	.065
Yes	76	15.3	10.5				
Extra bile duct resection							
No	185	26.3	13.3	.569			
Yes	10	30.0	9.9				
Lymphadenectomy							
No	182	24.9	13.3	.448			
Yes	13	46.2	12.1				
Other organ resection							
No	167	28.6	15.4	.015*		134 (0.84‐2.12)	.218
Yes	28	13.6	6.9				
Portal vein resection							
No	189	26.6	13.6	.278			
Yes	6	20.8	9.9				
Hepatic artery resection							
No	193	26.2	13.3	.855			
Yes	2	0.5	9.9				
IVC resection							
No	187	26.8	13.6	.257			
Yes	8	18.8	9.9				
Pringle's maneuver							
No	66	29.3	16.7	.401			
Yes	129	25.1	13.1				
Total hepatic vascular exclusion							
No	185	27.8	14.6	.001*		3.13 (1.46‐6.73)	.003*
Yes	10	0.0	6.3				
Allogenic red blood cell transfusion							
No	159	26.5	14.7	.575			
Yes	36	26.3	12.4				
Fresh frozen plasma transfusion							
No	134	23.7	13.0	.376			
Yes	61	31.8	14.5				
Postoperative complications							
No	126	26.9	13.3	.556			
Yes	69	25.8	13.6				
Histological variables							
Tumor histological grade							
Well differentiated	17	25.5	21.6	.970			
Moderate/poorly differentiated	155	28.8	13.3				
Margin status							
R0	114	34.2	21.6	<.001*		1.60 (1.11‐2.29)	.012*
R1	81	15.2	7.8				
Hepatic parenchymal histology							
Steatosis							
No	141	26.0	13.9	.859			
Yes	54	28.4	11.6				
Fibrosis							
No	189	26.1	13.3	.687			
Yes	6	44.4	24.4				
Sinusoidal obstruction syndrome							
No	177	27.4	14.5	.224			
Yes	18	18.1	7.8				

CI, confidence interval; DFS, disease‐free survival; IVC, inferior vena cava; MST, median survival time; PVE, portal vein embolization; RR, risk ratio; S1, caudate lobe; S2/3, left lateral section.

a Excluding 16 patients who died within 90 d after operation.

* *P* < .05.

**Table 4:** Univariate and multivariate analyses of variables predicting disease‐specific survival after right trisectionectomy

Variables	Total n = 195^a^	5‐y DSS (%)		MST (months)	Univariate	Multivariate	
					*P*‐value	RR (95% CI)	*P*‐value
Preoperative variables							
Gender							
Female	80	37.5		36.6	.999		
Male	115	38.1		41.7			
Age							
≤70 y	157	39.0		39.9	.380		
>70 y	38	31.5		38.1			
Preoperative PVE							
No PVE	182	39.1		40.2	.009*	1.74 (0.76‐3.97)	.190
PVE	13	0.0		24.6			
Preoperative chemotherapy							
No	111	44.0		43.9	.043*	1.20 (0.83‐1.74)	.338
Yes	84	27.5		36.7			
Neoadjuvant chemotherapy							
No	183	38.4		41.9	.065		
Yes	12	32.0		35.0			
Size of largest tumor							
≤100 mm	158	36.2		39.1	.231		
>100 mm	37	42.9		43.8			
No. of tumor							
Solitary	42	64.6		204.2	.001*	2.95 (1.68‐5.20)	<.001*
Multiple	153	30.0		32.8			
Type of liver metastasis							
Synchronous	107	36.7		39.9	.678		
Metachronous	88	38.9		39.1			
Intra‐ and postoperative variables							
S1 resection							
No	174	39.0		40.2	.042*	1.21 (0.64‐2.29)	.557
Yes	21	28.1		26.4			
S2/3 partial resection							
No	119	42.1		43.8	.023*	0.98 (0.67‐1.42)	.905
Yes	76	30.8		31.8			
Extra bile duct resection							
No	185	38.3		39.7	.702		
Yes	10	18.8		28.8			
Lymphadenectomy							
No	182	35.9		39.1	.222		
Yes	13	66.7		79.8			
Other organ resection							
No	167	38.0		39.7	.344		
Yes	28	38.8		48.6			
Portal vein resection							
No	189	39.0		39.6	.195		
Yes	6	0.0		39.7			
Hepatic artery resection							
No	193	37.6		39.6	.456		
Yes	2	50.0		48.6			
IVC resection							
No	187	38.5		39.9	.111		
Yes	8	17.5		39.7			
Pringle's maneuver							
No	66	36.6		37.8	.717		
Yes	129	38.1		40.0			
Total hepatic vascular exclusion							
No	185	39.1		40.0	.009*	3.77 (1.73‐8.20)	.001*
Yes	10	11.3		16.7			
Allogenic red blood cell transfusion							
No	159	37.2		39.7	.581		
Yes	36	39.5		38.5			
Fresh frozen plasma transfusion							
No	134	36.0		37.8	.651		
Yes	61	41.1		42.7			
Postoperative complications							
No	126	37.1		39.1	.990		
Yes	69	39.2		40.0			
Histological variables							
Tumor histological grade							
Well differentiated	17	37.6		39.7	.635		
Moderate/poorly differentiated	155	40.3		41.5			
Margin status							
R0	114	47.7		50.7	<.001*	1.92 (1.31‐2.81)	.001*
R1	81	22.9		20.8			
Hepatic parenchymal histology							
Steatosis							
No	141	36		38.5	.641		
Yes	54	42.6		43.9			
Fibrosis							
No	189	37.7		39.6	.949		
Yes	6	40		58.3			
Sinusoidal obstruction syndrome							
No	177	38.6		40	.339		
Yes	18	27.6		36.7			

CI, confidence interval; DSS, disease‐specific survival; IVC, inferior vena cava; MST, median survival time; PVE, portal vein embolization; RR, risk ratio; S1, caudate lobe; S2/3, left lateral section.

a Excluding 16 patients who died within 90 d after operation.

* *P* < .05.

### 3.4 Changes in outcomes over time and redo‐surgery for recurrence

**Figure 1:**
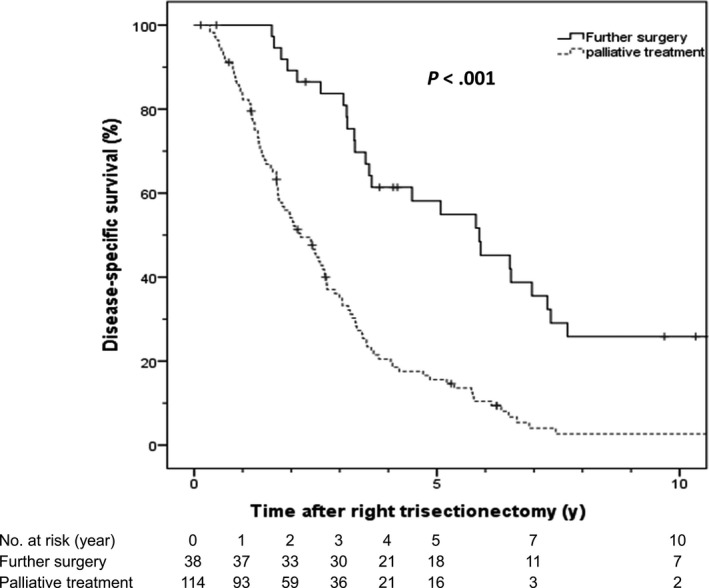
Disease‐specific survival following further surgery compared to palliative chemotherapy alone

To evaluate the impact of the learning curve and changes over the study period, patients were divided into three operative experience periods: first (n = 70; 33.2%), second (n = 70; 33.2%), and third (n = 71; 33.6%) period as described in Table 5. Median DFS was 33.8, 26.8 and 25.5 months from the first to the third period, *P* = .774. Frequency of further surgery for recurrent disease after RT increased steadily over time. Of the 211 patients who underwent RHT, 152 patients (72.0%) had disease recurrence, of whom 38 (25.0%) were eligible for further surgery. Eleven patients (7.2%) underwent a second repeat surgery, six (3.9%) patients had a third surgery and one (0.7%) patient a fourth repeat surgery with curative intent. Median interval from RHT to further surgery was 13.3 months; there was a median of 38.8 months to the second, 43.6 to the third, and 129.6 to the fourth surgery. Mortality after further surgery was zero. One or more redo liver resections were carried out in 31 patients (20.4%). One or more pulmonary surgeries including radiofrequency ablation were done in seven patients (4.6%). Two patients (1.3%) underwent other surgery for recurrent lesions (para‐aortic lymph node extirpation and pancreaticoduodenectomy). Of the 152 patients with recurrence, 114 patients (75%) had palliative chemotherapy alone. Five‐and 10‐year DSS were 58.1% and 25.8%, respectively, with a median DSS time of 70.5 months for the patients who underwent further surgery for recurrence and 15.6% and 2.7%, respectively, with a median DSS time of 26.4 months for those who had palliative treatment only (*P* < .001) (Figure 1).

**Table 5:** Changes in pre‐ and intraoperative management and postoperative outcomes according to the experience period

	Experience period, n (%)			*P*‐value
	1st period (n = 70)	2nd period (n = 70)	3rd period (n = 71)	
Age, years (range)	61 (36‐80)	61.5 (42‐84)	63 (25‐85)	.402
Preoperative chemotherapy	18 (25.7)	23 (32.9)	48 (67.6)	<.001*
Preoperative PVE	0 (0)	0 (0)	13 (18.3)	<.001*
Parenchymal liver damage	7 (10.0)	16 (22.9)	49 (69.0)	<.001*
Steatosis	5 (7.1)	14 (20.0)	38 (53.5)	<.001*
Fibrosis	2 (2.9)	3 (4.3)	1 (1.4)	.590
Sinusoidal obstruction syndrome	0 (0)	0 (0)	18 (25.4)	<.001*
S1 resection	4 (5.7)	7 (10.0)	14 (19.7)	.031*
S2/3 partial resection	19 (27.1)	30 (42.9)	31 (43.7)	.075
Vascular resection^a^	5 (7.1)	3 (4.3)	7 (9.9)	.436
Pringle's maneuver	33 (47.1)	43 (61.4)	61 (85.9)	<.001*
Total hepatic vascular exclusion	9 (12.9)	3 (4.3)	2 (2.8)	.023*
Allogenic red blood cell transfusion	28 (40.0)	12 (17.1)	4 (5.6)	<.001*
Fresh frozen plasma transfusion	47 (67.1)	30 (42.9)	8 (11.3)	<.001*
Morbidity	28 (40.0)	33 (47.1)	24 (33.8)	.271
Median hospital stay, days (range)^b^	11 (6‐34)	11 (4‐139)	9 (5‐71)	.119
90‐d mortality	9 (12.9)	5 (7.1)	2 (2.8)	.078
Further surgery for recurrence^c^	11 (21.6)	12 (24.0)	15 (29.4)	.645

PVE, portal vein embolization; S1, caudate lobe; S2/3, left lateral section.

a Including resections of portal vein, hepatic artery, and inferior vena cava.

b Excluding 15 patients who died in hospital.

c 51, 50, and 51 patients with recurrence in the 1st, 2nd, and 3rd periods, respectively.

* *P* < .05.

## 4 DISCUSSION

Several centers worldwide have reported their experience with RHT or LHT and extensions of RHT for a variety of indications in HPB malignancies. However, the existing evidence on this topic is scarce and limited to small numbers. Only a few studies for RHT and LHT have reported their experience in larger cohorts.^5^, ^7^, ^9^, ^13^, ^14^, ^26^, ^34^, ^35^, ^36^ This limited evidence might reflect the changing role of these demanding and extensive resections in daily HPB practice, with decreasing numbers being carried out today. This must be at least in part due to promising results for newly implemented surgical strategies such as TSH and ALPPS, along with oncological advances with better understanding of tumor biology resulting in increasing success with neoadjuvant treatment.

Extended resections have been associated with high morbidity and mortality rates.^37^ In 1988, Iwatsuki and Starzl reported their experience showing a mortality rate of 6.3% following right trisectionectomies in their series.^37^

In our original report of 275 patients undergoing trisectionectomies including various HPB malignancies, postoperative morbidity was 41%.^26^ Thirty‐day and 90‐day mortality rates were 7% and 8%, respectively.^26^ In this up‐to‐date study, morbidity among all RHT trisectionectomies carried out was 40.3%, and 30‐day and 90‐day mortality rates were 7.1% and 7.6%, respectively, following this procedure. In a separate analysis assessing morbidity and mortality rates over time, we have been able to show a tendency for reduced morbidity and mortality. These results have been confirmed by other groups.^36^ In their series of RHT, Matsumoto et al showed a morbidity rate of 27% and a mortality rate of 0%.^36^ These results seem to be comparable with outcomes following modern approaches such as TSH or ALPPS.^24^

In extensive liver resections, the size of FLR may have a crucial impact on postoperative morbidity and mortality. In general, 3%‐5% of patients may develop liver failure following liver resection. In the present study, the incidence of transient liver failure was 12% (mainly grade A or B according to the International Study Group of Liver Surgery) and reflects the magnitude of surgery. Controversy exists about the amount of liver volume essential to prevent liver failure following these operations. After RHT, FLR is variable but approximately 15%‐30% of the total liver volume is preserved. Recent studies have shown that FLR of less than 25%‐30% is predictive of hepatic dysfunction.^38^ Therefore, the use of PVE before trisectionectomy has been advocated to decrease postoperative morbidity and mortality and make these operations safer.^39^ When assessing PVE in the preoperative setting of right trisectionectomies, several studies have shown the importance of embolizing segment 4 to achieve sufficient hypertrophy.^40^, ^41^ Indeed, this has become part of our routine for patients with CRLM after chemotherapy before RHT and, in recent years, we have not experienced any postoperative mortality.

In the present study, multivariate analysis identified additional organ resection at the time of RHT and perioperative ARBC transfusion as independent predictors of postoperative morbidity. In this series, concomitant organ resection was carried out in 32 patients (15.2%) of the cohort. The diaphragm or large bowel was resected most frequently. The inferior outcome following multi‐organ resection most likely reflects a poor and aggressive tumor biology as well as advanced tumor stage.

In other studies, intraoperative blood loss and concomitant blood transfusion were identified as independent risk factors influencing morbidity, mortality and DSS.^9^, ^42^, ^43^, ^44^ This measurement indirectly displays the extent and quality of the surgery itself. A median of 4 units 1, 2, 3, 4, 5, 6, 7, 8, 9, 10, 11, 12, 13, 14, 15, 16, 17, 18, 19, 20, 21, 22, 23, 24, 25, 26, 27, 28, 29, 30, 31, 32, 33, 34, 35, 36, 37, 38, 39, 40 of red blood cells was transfused. Nearly 80% of patients did not require perioperative transfusion and there was a consistent decline in the need for transfusion over time. This may reflect improved surgical and anesthetic techniques as well as more conservative transfusion over time with higher thresholds for transfusion.

Our study further identified multiple tumors and R1 resections as independent predictors for recurrence and poor survival following liver resection for CRLM.^45^, ^46^, ^47^, ^48^, ^49^, ^50^ This corresponds to results of other studies.^51^, ^52^, ^53^ Sasaki et al clearly identified tumor size and number of CRLM as prognostic markers predicting outcome following resection of CRLM.^54^ Furthermore, our study identified TVE as an independent risk factor for poor survival. This might be due to advanced tumor stage when TVE was applied with invasion to the hepatocaval confluence or IVC. The radicality of surgery required, is associated with an increased potential for postoperative complications, this might be due to the fact that this type of surgery can potentially cause significant hemodynamic instability.^55^, ^56^, ^57^

Furthermore, in animal studies, TVE has been clearly linked to accelerated growth of hepatic micrometastases.^50^, ^58^ Indeed, nine out of 14 (64.3%) patients who required TVE had an R1 resection. In a separate analysis, a decrease of TVE over time was noted, again showing the increased experience with a higher caseload.

Repeat hepatic resection is technically challenging, with longer operative time than the initial surgery because of adhesions, altered anatomy, and fragile liver parenchyma as a result of chemotherapy, and it has rarely been reported following RHT.^15^, ^59^, ^60^, ^61^, ^62^, ^63^ However, in this series, repeat resection showed better 5‐ and 10‐year DSS of 58.1% and 25.8%, respectively, compared to DSS of 15.6% and 2.7% in patients undergoing palliative chemotherapy only. Furthermore, there was no postoperative mortality among patients who underwent repeat resections and none developed liver failure in the further postoperative course.

A limitation of the present study is the incomplete data on chemotherapy. As a result of this limitation, a detailed analysis of the role of chemotherapy in this treatment algorithm was not possible. Only a relatively small proportion of patients received neoadjuvant therapy for downsizing, but the proportion in whom downsizing strategies failed was not captured in the data set.

## 5 CONCLUSION

Left hepatic trisectionectomy and RHT are technically demanding liver resections with a high risk for perioperative morbidity and, in the past, also mortality. Our data show these risks are reducing with experience, better patient selection, and the more liberal use of PVE. LHT and RHT remain relevant for many situations but innovation in surgery and neoadjuvant treatments inevitably mean that the role of these challenging operations is decreasing.

## DISCLOSURE

Conflicts of Interest: Authors declare no conflicts of interest for this article.
